# Gas Sensor Array Fault Diagnosis Based on Multi-Dimensional Fusion, an Attention Mechanism, and Multi-Task Learning

**DOI:** 10.3390/s23187836

**Published:** 2023-09-12

**Authors:** Pengyu Huang, Qingfeng Wang, Haotian Chen, Geyu Lu

**Affiliations:** State Key Laboratory of Integrated Optoelectronics, College of Electronic Science and Engineering, Jilin University, Changchun 130012, China

**Keywords:** gas sensor array, fault diagnosis, deep learning, attention mechanism, multi-task learning

## Abstract

With the development of gas sensor arrays and computational technology, machine olfactory systems have been widely used in environmental monitoring, medical diagnosis, and other fields. The reliable and stable operation of gas sensing systems depends heavily on the accuracy of the sensors outputs. Therefore, the realization of accurate gas sensor array fault diagnosis is essential to monitor the working status of sensor arrays and ensure the normal operation of the whole system. The existing methods extract features from a single dimension and require the separate training of models for multiple diagnosis tasks, which limits diagnostic accuracy and efficiency. To address these limitations, for this study, a novel fault diagnosis network based on multi-dimensional feature fusion, an attention mechanism, and multi-task learning, MAM-Net, was developed and applied to gas sensor arrays. First, feature fusion models were applied to extract deep and comprehensive features from the original data in multiple dimensions. A residual network equipped with convolutional block attention modules and a Bi-LSTM network were designed for two-dimensional and one-dimensional signals to capture spatial and temporal features simultaneously. Subsequently, a concatenation layer was constructed using feature stitching to integrate the fault details of different dimensions and avoid ignoring useful information. Finally, a multi-task learning module was designed for the parallel learning of the sensor fault diagnosis to effectively improve the diagnosis capability. The experimental results derived from using the proposed framework on gas sensor datasets across different amounts of data, balanced and unbalanced datasets, and different experimental settings show that the proposed framework outperforms the other available methods and demonstrates good recognition accuracy and robustness.

## 1. Introduction

With the development of sensor technology, gas sensor arrays are playing an increasingly important role in machine olfaction applications such as environmental monitoring [1], gas quality detection [2], food quality control [3], and medical diagnosis [4]. A machine olfactory system (e.g., an electronic nose) identifies gases by analyzing the data returned from a gas sensor array using pattern recognition methods. Metal-oxide semiconductor (MOX) gas sensors are widely used because of their low cost, high sensitivity, and fast response times. For example, Ref. [5] proposed an ultrasensitive gas sensor established on hollow tungsten trioxide-nickel oxide nanoflowers, which had a fast response time and outstanding gas sensitivity. A pattern recognition algorithm is an important part of electronic noses in analyzing gas characteristics, and this type of algorithm is often used to classify gas mixtures. Common methods include principal component analysis (PCA) [6], artificial neural networks (ANNs) [7], and convolutional neural networks (CNNs) [8]. These methods are implemented on the basis of data from healthy sensors. A gas sensor array is the source of the machine olfactory system used to obtain measured gas/odor information, and its measurement quality significantly determines the overall performance of the system.

However, the MOX gas sensor array inevitably suffers from external interference (corrosive gas influence, dust adhesion, temperature, and humidity changes) or self-failure (aging, poisoning, and damage to gas sensing materials) during operation. Once the sensor fails, the application of inaccurate measurements will lead to decreases in the accuracy and reliability of the classification results or even complete errors [9]. Therefore, appropriate fault diagnosis algorithms must be employed to monitor the abnormal states of the gas sensor array (fault detection), identify fault types (fault identification), and locate faulty gas sensors (fault localization).

With the advancement of automation and integration in modern industry, there has been an increasing demand for the reliability and safety of related equipment. Fault diagnosis and health management techniques have been used in various industrial applications in recent years, such as the physics-informed residual network (PIResNet) for rolling element bearing fault diagnostics [10], digital twin-driven intelligent assessment of gear surface degradation [11], and a novel vibration-based prognostic scheme for gear health management in the surface wear progression of the intelligent manufacturing system [12]. All these methods can provide effective solutions for health management and the predictive maintenance of working systems in industrial processes. In addition, there has been a surge in research focused on monitoring the fault status of sensors commonly used in modern industry. These studies aim to enhance the reliability of measurement signals. Currently, sensor fault diagnosis is primarily based on data-driven methods [13]. This approach can directly discover and analyze hidden information in the training data instead of building complex mathematical models to describe the fault characterization principle in advance. Machine learning (ML) and deep learning (DL) are the most frequently used data-driven methods for fault diagnosis. Traditional ML methods are widely utilized for early sensor fault diagnosis. Common methods include the k-nearest neighbor algorithm [14], support vector machine [15], PCA [16], and ANN [17]. However, as the size and complexity of the processed samples increases, ML methods have difficulty determining the appropriate hyperparameters for feature extraction, which is a challenging task without sufficient prior knowledge.

With improvements in computer-processing capabilities, DL-based fault diagnosis methods have emerged as alternatives to traditional ML methods. DL-based methods can automatically extract classification features from large-scale data, overcoming the limitations of traditional feature extraction [18,19]. Therefore, deep learning is widely used to process large amounts of complex sensor data. Common DL-based approaches include CNN, long short-term memory (LSTM), and generative adversarial networks (GANs). To overcome the tediousness of ML in data preprocessing, many DL-based methods convert the original data into a two-dimensional (2D) image format suitable for CNN processing. In [20], a deep CNN-based diagnostic model was constructed for the fault classification of the sensors and actuators of robot joints. A CNN fault identification approach based on the time–frequency characteristics of UAV sensor signals was proposed in [21]. Both methods improve recognition accuracy compared with traditional ML methods. With the development of CNNs, improved models have been applied in sensor fault diagnosis. Ref. [22] described a fault detection method for aeroengine sensors based on the Inception–CNN model. Compared with an ordinary CNN, this model can extract more sensor information at different scales to increase the diagnostic accuracy of fault state detection. LSTM is also an effective method for processing sensor data, as it is capable of extracting one-dimensional (1D) temporal dependencies directly from sequence data. Ref. [23] used an LSTM model for voltage sensor fault identification in battery energy storage systems in 1D temporal dimensions. A method based on a 1D CNN and LSTM was proposed for the fault identification and recovery of Hall sensors in [24]. This method further extracts features from a temporal perspective and exhibits high diagnostic accuracy. GANs are commonly applied to generate samples similar to real samples through their adversarial structures, which usually consist of a generator and a discriminator [25]. Additionally, GANs have also been employed in various fault diagnosis tasks due to their feature extraction capabilities. In [26], a fault detection model was proposed based on a one-dimensional residual GANomaly network, which effectively enhanced training efficiency and diagnostic accuracy. Ref. [27] developed a fault identification method based on Bi-LSTM, GAN, and autoencoder (AE). This method utilizes the reconstructed features obtained by GAN and AE to identify sensor faults with excellent performance.

Several methods based on DL have also been proposed for gas sensor fault diagnosis. Sun et al. [28] employed an improved CNN and random forests to classify the fault types of gas sensors. In [29], the authors used transfer learning combined with LeNet-5 for gas sensor fault identification. A method that combines a CNN and deep convolutional GAN to address dataset imbalances in fault type classification was proposed in [30]. These methods employed the basic CNN structure for feature extraction when classifying fault types in a sensor array comprising a limited number of gas sensors.

Although the models proposed above have achieved good results, some shortcomings remain in dealing with increasingly high-dimensional complex data for gas sensor fault diagnosis. (1) These methods can only extract features from a single dimension and ignore potential features from other perspectives, which limits the feature extraction capabilities of such models in complex situations. (2) These models cannot selectively focus on or ignore features based on their level of contribution to the classification results, which may fail to capture key features and waste considerable amounts of computational resources. (3) These diagnostic methods are trained for only a single diagnostic task, which loses potential correlation features between samples, limiting the diagnostic performance and utilization of the extracted features and the need to perform multiple diagnosis tasks. 

The multi-feature fusion strategy is a solution for capturing the comprehensive features of samples across different scales [31,32,33]. Three types of multi-feature fusion algorithms are commonly used nowadays. The first involves converting the original samples into frequency–domain signals, time–frequency diagrams, or amplitude-modulated–frequency-modulated components using techniques such as Fourier transform, wavelet transform, and empirical modal decomposition. Feature extraction and fusion are subsequently performed on these different forms of signals [34,35]. However, this approach is limited in obtaining additional feature information when applied to the smoothed data from gas sensors, and it also increases the complexity of the entire process. The second approach involves utilizing different deep learning models to extract and fuse features from the same perspective. In [36], a fault prediction model based on a hybrid deep neural network model was proposed, and Qian et al. [37] developed a parallel deep learning framework based on multiple models for the abnormal prediction of data in industrial production. Both of these methods use a parallel structure consisting of a 1D CNN and LSTM to capture temporal features. However, this type of approach tends to ignore features that exist in other perspectives. The third approach involves fusing features from different perspectives or scales. CNNs with convolutional kernels of different sizes were used in [32,33] to extract multi-scale features simultaneously. In [38], LSTM and CNN were used to extract both the temporal and spatial features of the samples used.

Although these methods improve the amount of information for feature extraction, they are prone to ignore some decisive feature details because they are unable to select features that actively contribute significantly to classification for learning. Reportedly, a combination of network models and attention mechanisms can effectively improve the feature extraction quality. In [39], the authors proposed a fault type classification strategy for aircraft attitude sensors using the RepVGG and SENet attention mechanisms. Ref. [40] developed an attention mechanism for deep residual shrinkage networks using a fault identification model. This mechanism can effectively improve the identification of fault features using a model in a signal with noise. Unfortunately, each of these methods [29,30,31,32,33] was only trained for a single task, which limits their recognition accuracy and efficiency. Multi-task learning is a paradigm in machine learning that can process multiple tasks simultaneously and improve the generalization performance and classification accuracy of a model [41]. In [42], multi-task learning was applied to the fault type classification and fault level detection of wind turbine blades and obtained better results than single-task learning. Similarly, in another study, multi-task learning was used to diagnose bearing faults and proved capable of elucidating the type and size of each fault in parallel [43]. These methods demonstrate the advantages and feasibility of multi-task learning for fault diagnosis.

Above all, the existing methods for the fault diagnosis of gas sensors extract features from a single dimension, ignoring the potentially discriminative features in other dimensions. Additionally, the single-task learning methods for three diagnostic tasks require diagnostic models to be trained separately, which increases complexity and limits diagnostic accuracy. Therefore, in this paper, a novel network based on multi-dimensional feature fusion, attention mechanism, and multi-task learning (MAM-Net) for gas sensor array fault diagnosis is proposed. The main contributions of this paper are as follows:

(1) A multi-dimensional feature fusion method integrating a residual network (ResNet) and Bi-LSTM is proposed. Deep and comprehensive features can be extracted by fusing the 2D spatial features and 1D temporal features of samples for the fault diagnosis of a gas sensor array.
(2) A ResNet equipped with convolutional block attention module (CBAM) is proposed for the 2D feature extraction of gas sensor data to capture and refine important fault features more effectively, and the diagnostic accuracy of the model is further improved.
(3) A multi-task learning module was designed for gas sensor fault detection, fault identification, and fault localization. This approach can fully utilize the extracted comprehensive features to perform the three tasks in unison. The diagnostic accuracy can be improved by parameter sharing and the mutual promotion of simultaneous training between related tasks.

The remainder of this paper is structured as follows: In Section 2, the theoretical background for our proposed framework is described. Section 3 provides a systematic description of the proposed framework and its internal modules. Section 4 introduces the datasets used in our experiments. Section 5 presents the results and analysis. In Section 6, we discuss and analyze the performance of the proposed method in other cases. Finally, Section 7 states the conclusions of this study.

## 2. Theoretical Background

### 2.1. ResNet

As an updated version of a traditional CNN, ResNet aims to avoid gradient disappearance and explosion as the depth of the neural network increases [44]. A ResNet is generally built using several residual blocks with a mapping function designed as follows:

(1) $$H\left(x\right)=F\left(x\right)+x,$$

where x is the residual block input, $F\left(x\right)$ is the residual mapping, and $H\left(x\right)$ is the residual block output. The constant mapping x connects the residual block input and output, which facilitates backpropagation of the loss function and the optimization of the model parameters [45]. 

Due to the structure and advantages of ResNet, this network can easily fit various complex data in two dimensions and is highly effective for feature extraction during fault diagnosis. In [46], the authors used ResNet for the feature extraction of the wavelet coefficients of the original letter decomposition from 2D to identify wind turbine gearbox faults. Ref. [47] converted the original signal into an image using a Markov transfer field and subsequently applied ResNet to extract 2D features to achieve the classification of rolling bearing health conditions. Therefore, in this study, ResNet with an attention mechanism was designed as the 2D feature encoder for the proposed framework.

### 2.2. CBAM

**Figure 1:**
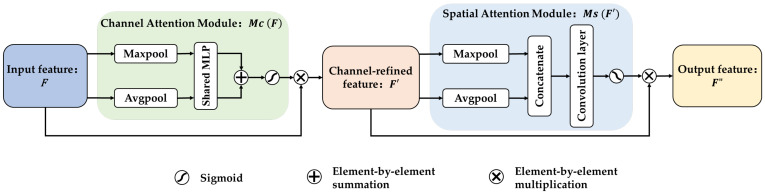
Basic structure of CBAM.

The CBAM is a lightweight attention module [48]. It consists of a channel attention module (CAM) and a spatial attention module (SAM). Its structure is illustrated in Figure 1. Within the intermediate feature maps of a network, the CBAM can sequentially infer attention maps along the channel and spatial dimensions to obtain refined features [49].

In the CAM, the intermediate feature map F as input is pooled by global maximum pooling and global average pooling (GAP) based on the channel. The resulting vectors are then sent to the fully connected (linear) layer separately for information sharing and are stitched together to obtain the channel attention map. Subsequently, the channel attention activated by the sigmoid function is multiplied times F to obtain the channel refinement feature map $F^{\prime }$. In the SAM, $F^{\prime }$ is pooled by global maximum and average according to space, and the resulting 2D vectors are connected. A spatial attention map is then generated by convolving and activating the combined vectors. Finally, the spatial attention is multiplied times $F^{\prime }$ to obtain the output feature map $F^{\prime \prime }$ of CBAM. This process is represented by the following equations:

(2) $$F^{\prime }=Mc\;\left(F\right)\;⊗\;F,$$

(3) $$F\prime \prime =Ms\;\left(F^{\prime }\right)\;⊗\;F^{\prime },$$

where F, $F^{\prime }$, and F″ represent the input feature map, feature map of the channel attention output, and feature map of the spatial attention output, respectively. $Mc\;\left(F\right)$ is the output of the CAM, and $Ms\;\left(F^{\prime }\right)$ is the output of the SAM. ⊗ denotes element-by-element multiplication.

Due to the attention mechanism, CBAM can allocate weights to different feature maps, helping the model focus on positions with outstanding features and suppress the regions that contribute little to the results, which improves the recognition effectiveness of the feature extraction model. Ref. [50] employed CNN and CBAM to improve the accuracy for classifying rolling bearing fault types. In [51], the authors used CBAM and ResNet for fault detection in reciprocating compressors to enhance the model’s representation of key features. Therefore, we introduced this module into ResNet in this study to improve gas sensor array fault diagnosis.

### 2.3. Bi-LSTM 

**Figure 2:**
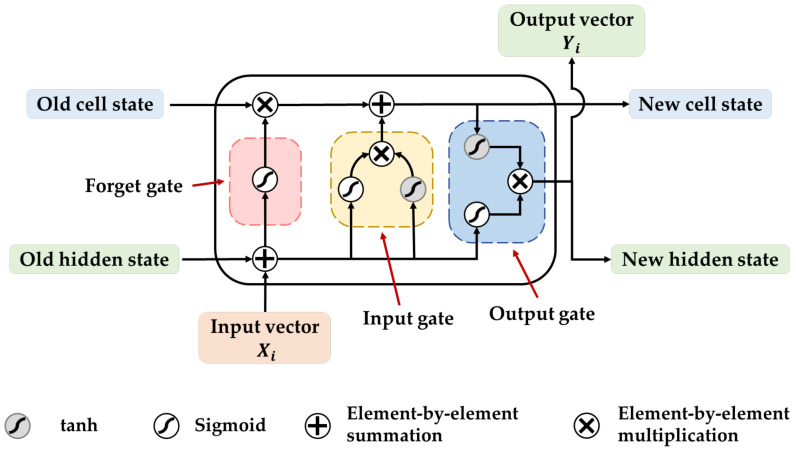
Basic structure of LSTM.

LSTM is a derivative of a recurrent neural network (RNN). It learns features by considering the order of sequential data, thereby overcoming the problems of gradient explosions and vanishing gradients in RNN by capturing long-distance dependencies [52,53]. LSTM consists of three gate cells (forget, input, and output gates) and a memory cell (cell state), as shown in Figure 2. The cell state runs across all LSTM cells to transfer information over long distances. The input and forget gates decide whether to retain or discard information about the cell states, and the output gate generates the output vector $Y_{i}$ based on the cell state and input vector $X_{i}$. This architecture enables it to maintain important features effectively during long-term processes [27].

Bi-LSTM is an extension of LSTM. It comprises two LSTM that can simultaneously process time-series information in the forward and reverse directions. This approach effectively increases the amount of information available to the network [54] and enhances the understanding of fault features in the model [55]. Therefore, we utilized Bi-LSTM as a 1D feature-encoding module in this study to complement the 2D features extracted by ResNet, thus enhancing the feature extraction capability of the model.

## 3. Proposed Method

**Figure 3:**
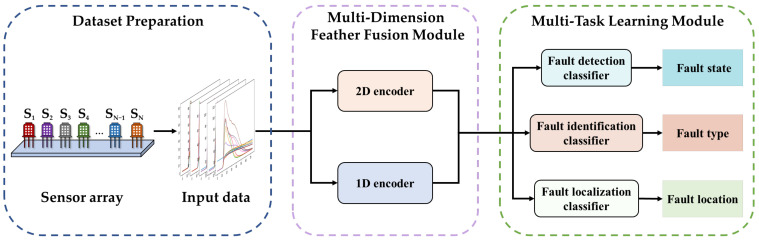
Framework of the proposed method.

This section introduces the proposed method for gas sensor array fault diagnosis in detail, and its structure is illustrated in Figure 3. The sensor array is composed of the gas sensors S_1_–S_N_. The data matrix, which consists of the output value of each sensor during gas detection, is preprocessed to transform it into a 2D image (the input data of the proposed model). The outputs of this method are labels with multiple fault descriptions of the sensor array.

The proposed MAM-Net comprises two main parts: a multi-dimensional feature fusion module and multi-task learning module. The multi-dimensional feature fusion module contains 1D and 2D encoders, which are used to obtain sufficient information from the training data to improve the accuracy of fault diagnosis. The multi-task learning module is utilized to conduct fault detection, fault identification, and fault localization and then outputs the status, type, and location of the fault simultaneously. The details of each module are as follows.

### 3.1. Multi-Dimensional Feature Fusion Module

**Figure 4:**
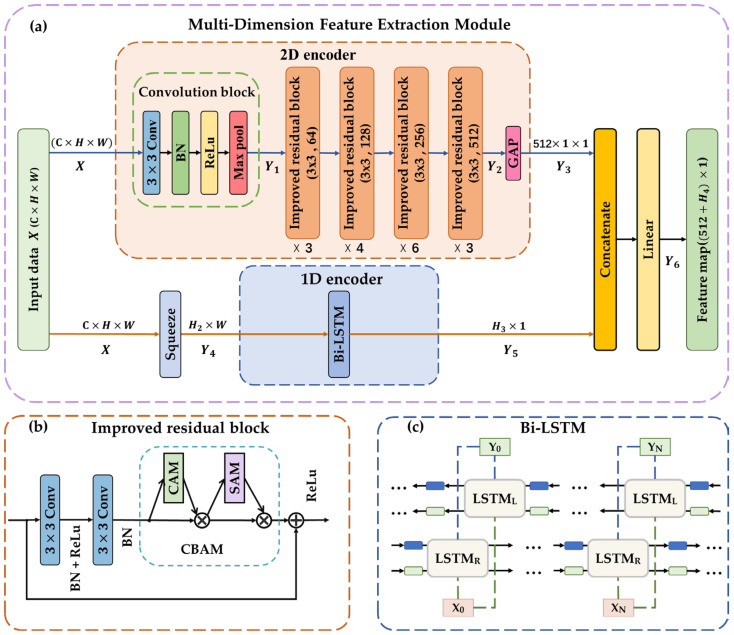
Schematic of the developed multi-dimensional feature fusion method. (**a**) Architecture of multi-dimensional feature fusion module. (**b**) Internal structure of the improved residual block in the 2D encoder in (**a**). (**c**) Internal structure of the Bi-LSTM in the 1D encoder in (**a**).

The proposed multi-dimensional feature fusion module consists of two parallel paths—1D and 2D encoders—and concatenate and linear layers, as shown in Figure 4a. The two encoders are utilized to encode fault features in the temporal and spatial dimensions in parallel. The concatenate and linear layers are employed to fuse the feature information from the two dimensions.

In the 2D encoder, ResNet is used as the backbone network to extract the 2D spatial features of the faulty data. The network consists of a convolutional block, multiple improved residual blocks, and a GAP layer, as illustrated in Figure 4a.

To satisfy the requirements of the multi-dimensional feature fusion module in the proposed method for the image format of the sensor array data, the original input data are converted into a 2D data structure, $X\in R^{C\times H\times W}$, where C, H, and W correspond to the number of channels, height, and width of the image, respectively. H also represents the number of sensors, W denotes the period length of the sample, and C = 1. The input data *X* first proceed through a convolutional layer (kernel size = 3 × 3), batch normalization (BN) layer, ReLU activation function, and max pooling layer in the convolution block to obtain the basic feature map $Y_{1}\in R^{C_{1}\times H_{1}\times W_{1}}$ ($C_{1}$, $H_{1}$, and $W_{1}$ are the corresponding sizes after convolution). Subsequently, $Y_{1}$ is fed into multiple stacks of improved residual blocks to obtain additional fault characteristics. The improved residual block is formed by adding CBAM to the traditional residual block. The structure is shown in Figure 4b. In this residual block structure, the feature map is first processed using two convolutional layers, two BN layers, and one ReLU activation function layer for the overall features. Subsequently, the CBAM is employed to enhance the representation of effective features and suppress the interference of invalid features by changing the weight parameters of different feature information in the feature map. This approach enables the model to focus more on the locally important features of the data, such as the moment of the peak in the spike fault and the moment at which the return value approaches zero in the broken circuit fault. Thus, a feature map $Y_{2}\in R^{C_{2}\times H_{2}\times W_{2}}$ can be obtained after several improved residual blocks. Finally, GAP is used to summarize all fault feature information extracted by the 2D encoder. GAP can efficiently represent the classification information contained in each channel by averaging the $H_{2}\times W_{2}$ feature values in each channel (i.e., $C_{2}$ channels) of feature map $Y_{2}$. Then, the vector formed by the obtained $C_{2}$ averages is employed as feature map $Y_{3}$.

The Bi-LSTM is the main body of the 1D encoder, as shown in Figure 4c, and it can extract fault features from sensor data in the temporal dimension. The channel dimension in X is removed by the squeeze layer to obtain feature map $Y_{4}\in R^{H\times W}$ (where *H* denotes the number of sensors and also represents the number of channels in 1D feature extraction). The network learns the time-sequence features in a manner similar to natural language processing. It considers the relationships among multiple sensor data points at each sampling point as the embedding of information for that moment. The relationships among different moments of information are used as features. Compared to the 2D encoder, which captures the spatial relationships between data in a sample as features, this network focuses on the backward and forward dependencies of time-series data as features. This approach overcomes the limitations of 2D feature extraction. For example, high similarities exist between the local spatial features of spike faults and noise faults, and one cannot distinguish the two types of faults well by only using a 2D encoder for feature extraction. However, the characteristics of these two fault types differ significantly from one another from a temporal perspective. Therefore, with the assistance of a 1D encoder, the model can perform well in distinguishing fault types. The temporal feature map $Y_{5}$ is obtained after Bi-LSTM processing.

Finally, the model concatenates the 1D feature map $Y_{5}$ with the 2D feature map $Y_{3}$ along the feature dimension and passes it to a linear layer to integrate the information and obtain the final feature map $Y_{6}$. This feature map can provide more adequate and effective fault feature information for subsequent multi-task learning models.

### 3.2. Multi-Task Learning Module 

**Figure 5:**
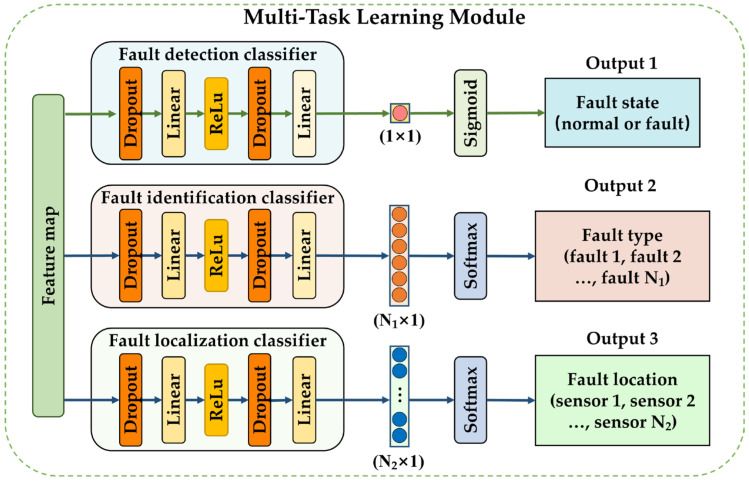
Structure of the multi-task learning module.

As shown in Figure 5, the multi-task learning module consists of three different classifiers: a fault state classifier, a fault type classifier, and a fault location classifier. It obtains multi-dimensional feature maps from the multi-dimensional feature fusion module and passes them to different classifiers. Each classifier has the same structure with different parameter settings; each contains two dropout layers, two linear layers, and a ReLU activation function. The dropout layers are used to limit the number of participating training neurons to avoid overfitting. The linear layer can be utilized to establish linear functions to fit the relationship between sample labels and features by adjusting the weights. The ReLU activation function can enhance the generalization ability of each classifier by introducing nonlinear relationships between linear layers.

Unlike single-task learning, multi-task learning can calculate the loss values of multiple classification tasks in a single training session and update the network parameters based on these loss values. This approach enables several related diagnostic tasks to share feature information, improving the diagnostic accuracy and generalizability of each classification task.

#### 3.2.1. Fault Detection Classifier

This classification model is designed to determine whether a sample is faulty and can categorize samples into two classes, namely, “normal” and “faulty”, with corresponding labels of 0 and 1, respectively. Because this classification task is binary, the final linear layer of the classifier is connected to a sigmoid activation function to transform the model output into a range from 0 to 1. This approach enables the probability of a sample being “normal” to be calculated, where a probability of 0.5 is used as the threshold. Subsequently, binary cross-entropy is utilized as a loss function of fault detection to calculate the loss values $Loss_{1}$ based on the output values of the classifier and its corresponding labels. Finally, the network parameters are updated using the Adam optimizer. The loss function is defined as follows:

(4) $$Loss_{1}=-\frac{1}{batch\;size}\overset{batch\;size}{\underset{i=1}{\sum }}\left[y_{i}\log\left(p_{i}\right)+\left(1-y_{i}\right)\log\left(1-p_{i}\right)\right],$$

where the *batch size* represents the number of samples in a single training epoch, $y_{i}$ represents the label of the ith sample, and $p_{i}$ is the probability that the result predicted by the model is the true label.

#### 3.2.2. Fault Identification and Localization Classifier

The fault type and location classifiers differ from the fault detection classifier in that they are both multi-classification classifiers. The fault identification classifier can classify the samples into $N_{1}$ types according to the fault type, such as no fault, broken circuit fault, spike fault, or noise fault. These fault types correspond to labels ranging from 0 to $N_{1}$. For a gas sensor array consisting of $N_{2}$ sensors, the fault location, as the output of the fault localization classifier, can be labeled from 0 to $N_{2}$, where 0 indicates that no fault has occurred in the array. 

To perform these multiple classification tasks, the classifier first normalizes the model output vector using the softmax activation function to obtain the vector $\hat{y}$. Values ranging from 0 to 1 within $\hat{y}$ represent the probabilities of different classes, and the class corresponding to the maximum value is the prediction result of the model. Subsequently, the cross-entropy loss function is used to calculate the loss values between the model outputs $\hat{y}$ and the true label y of the samples to update the parameters of the model. 

The loss values for fault identification and localization are calculated using the cross-entropy loss functions $Loss_{2}$ and $Loss_{3}$ and can be mathematically defined as follows:

(5) $$Loss_{2}=Loss_{3}=-\frac{1}{batch\;size}\overset{batch\;size}{\underset{j=1}{\sum }}\overset{C}{\underset{i=1}{\sum }}y_{ji}\cdot \log{\hat{y}}_{ji},$$

where $y_{ji}$ represents the true value of the ith class for the *j*th sample in a batch; ${\hat{y}}_{ji}$ is the corresponding predicted value; and *C* represents the number of classes, which, in fault identification and localization, is $N_{1}$ and $N_{2}$, respectively.

### 3.3. MAM-Net Model Training

The training of the MAM-Net is a process of constantly optimizing the loss values corresponding to the three classifiers. First, the loss values $Loss_{1}$, $Loss_{2}$, and $Loss_{3}$ are calculated based on the multiple fault labels of the input samples with the predictions of the model. Subsequently, a backpropagation (BP) algorithm is used to calculate the gradient from the output layer to the input layer based on three sequential loss values. Finally, the Adam optimizer is employed to update the parameters of the model based on the gradient information calculated in the model to minimize the loss function. Due to the application of the multi-task learning method, Adam can update the model based on three types of gradient information simultaneously during training. This method realizes parameter sharing and cross-task learning across three tasks.

## 4. Dataset Preparation

### 4.1. Dataset Description

**Figure 6:**
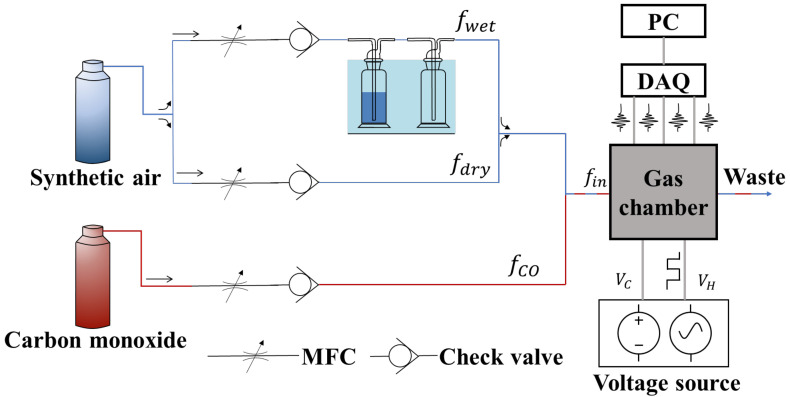
Experimental setup [56] for data collection of gas sensors.

A carbon monoxide gas sensor dataset collected by Javier Burgués et al. [56,57] was used in the experiments. This dataset was obtained from 14 temperature-modulated MOX gas sensors, including 7 SB-500-12 units from Nissha FIS and 7 TGS3870-A04 units from Figaro Engineering. The experimental setup for the data collection of gas sensors is shown in Figure 6. The dataset was obtained by exposing the sensor array to mixtures of carbon monoxide and humid synthetic air in a gas chamber. During the experiment, the heating unit inside the sensor was voltage-modulated with 20 s and 25 s interval cycles, as recommended by the manufacturer. The entire measurement process took 3 weeks. 

**Figure 7:**
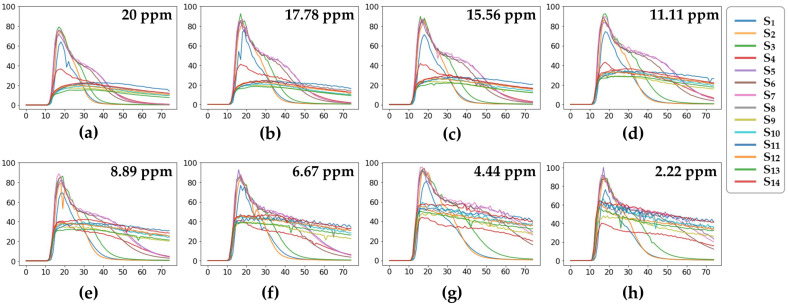
Output curves from the 14 gas sensors (S_1_–S_14_) for some samples with CO concentrations of (**a**) 20 ppm, (**b**) 17.78 ppm, (**c**) 15.56 ppm, (**d**) 11.11 ppm, (**e**) 8.89 ppm, (**f**) 6.67 ppm, (**g**) 4.44 ppm, and (**h**) 2.22 ppm.

To ensure that the data format was consistent, we used samples with the same period length and stable gas concentration as those used in the training data through data preprocessing. The final sample size obtained was 75 × 14 (where 75 denotes the period length and 14 represents the number of sensors). Figure 7 shows the output curves of the 14 gas sensors at different gas concentrations for some of the samples.

To achieve accurate fault diagnosis predictions, balanced data should be trained using deep neural networks [30]. Therefore, in this study, the number of samples in the preprocessed dataset was adjusted to ensure that the amount of data for each sensor at various concentrations was consistent. The number of samples for each gas concentration was adjusted to 266 (14 × 19, where 14 represents the number of sensors and 19 represents the number of samples allocated to each sensor at each concentration). The diagnosis performance in the case of imbalanced data is discussed in Section 6.

### 4.2. Fault Injection

Fault injection is a suitable method of testing the validity of a diagnostic model. The fault injection method can be utilized to test the efficacy of a model by adding fault features to sensor measurements to simulate the actual fault data. Subsequently, the fault occurrence time and fault intensity can be randomly changed via fault injection to generate a more representative fault dataset and test the generalization ability of the model [58].

In the experiments, we considered five types of sensor faults based on the existing studies on sensor fault characteristics: broken circuit, bias, spike, noise, and gain faults [19,59]. The characteristics of each fault type can be described as follows:

- Broken circuit fault: The value returned by the gas sensor drops to zero and stops changing because of a circuit break or short circuit in the system.
- Bias fault: The output value is stabilized around a fixed value due to the reaction-sensitive unit of the semiconductor gas sensor with the heating wire off.
- Spike fault: The output value appears as a pulse value because of an abnormal voltage spike pulse in the sensor circuit.
- Noise fault: The output values appear irregular and strongly disturbed because of external disturbances.
- Gain fault: The output value has a constant ratio to the ideal value because of internal circuit issues.

**Figure 8:**
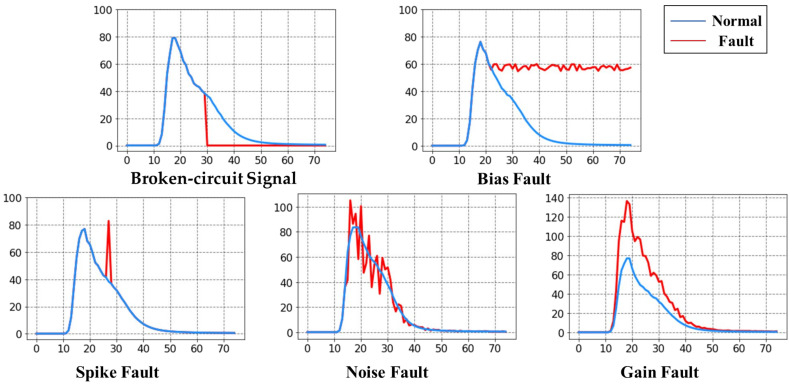
Example comparisons between various types of faults and normal signals.

The relationship between the original health data $x\left(t\right)$ and fault data $y\left(t\right)$ after the fault injection is summarized as follows:

(6) $$y\left(t\right)=Kx\left(t\right)+B\left(t\right),$$

where K and $B\left(t\right)$ are parameters for which the settings for each fault type are listed in Table 1. Examples of the corresponding data curves after fault injection into the health dataset and normal signals are shown in Figure 8. 

To compare the fault diagnosis performance for different amounts of data, we divided the dataset obtained after fault injection into three datasets while maintaining data balance, which, from largest to smallest, were Dataset1 (Ds1), Dataset2 (Ds2), and Dataset3 (Ds3). Ds1 contained 12,768 samples, Ds2 contained 6720 samples, and Ds3 contained 3360 samples. The numbers of occurrences of the various fault types in each dataset are listed in Table 2. Finally, each synthetic fault dataset was divided into training, validation, and testing datasets in a ratio of 8:1:1.

## 5. Experimental Results

A series of two types of experiments were conducted to validate the effectiveness and superiority of the proposed model. 

One type of experiment aims to explore the optimal network structure under different components, combinations, and module parameters in our proposed MAM-Net structure and to verify the advantages of each component module (2D-ResNet 34, ResNet with CBAM, multi-dimensional feature extraction based on ResNet with CBAM and Bi-LSTM, multi-task learning module) in the proposed model. The type of experiments is carried out through the following three comparisons: (1) comparing the performance of the 2D encoder with different network depths, dimensions, and attention modules; (2) comparing the effect of multi-dimensional feature extraction with that of single-dimensional feature extraction on the model recognition accuracy; (3) comparing the classification effect of multi-task learning with that of single-task learning.

The other type of experiment aims to demonstrate the superiority of the proposed MAM-Net by conducting comparisons with existing gas sensor fault diagnosis methods on three fault diagnostic tasks.

All experiments were performed on a computer (Intel Xeon E5-1603 V4 CPU and NVIDIA GeForce GTX 1080Ti GPU) on a PyTorch platform built with a Jupyter notebook. During the training of the model, the batch size and iteration epochs were set to 32 and 50, respectively. The test results were the averages of the best accuracies obtained after training the model five separate times on the training set.

### 5.1. Two-Dimensional Encoder Performance Comparison

To validate the advantages of the depth, dimension, and attention mechanism of ResNet used by the 2D encoder in the proposed model, we conducted the following experiments.

#### 5.1.1. Performance Comparison of ResNet with Different Depths and Dimensions

In the field of machine vision, the recognition accuracy of a model can typically be improved by increasing the network depth [60]. The recognition accuracies (Acc) of the ResNet with different depths (Dep) in the three classification tasks for Ds1, Ds2, and Ds3 are listed in Table 3. ResNet34 exhibits good classification performance for all three datasets. Although ResNet50 can perform the best on Ds1, which has a larger amount of data, its accuracy gradually decreases as the amount of data decreases and eventually falls below that of ResNet34. Due to the challenges in collecting substantial amounts of fault data in real-world situations, we selected ResNet34 as the main body of the 2D encoder. 

Furthermore, ResNet, which is currently employed in sensor arrays, typically utilizes 1D or 2D convolutional kernels; the 1D convolutional kernel version replaced all the 3×3 convolutional kernels of the 2D version into a 3 × feature-dimension, and the pooling operation was transformed from 2D to 1D [61]. In contrast, the average diagnostic accuracy of the 1D convolutional version was lower than that of the 2D version, as shown in Table 3. Therefore, the feature extraction module formed by the 2D version of ResNet has stronger feature capturing capabilities.

#### 5.1.2. Performance Comparison of Different Attention Modules

To discuss the advantages of using a CBAM in the proposed method, we compared the enhancement effects of different attentional modules on the 2D encoder feature learning ability in this experiment. The average recognition accuracies of ResNets with different attention mechanisms, including SENet [52], DRSN [29], and CBAM [37] for fault detection, fault identification, and fault localization on Ds1, Ds2, and Ds3, respectively, are listed in Table 4.

The results indicated that the combination of ResNet and CBAM performed the best in the classification tasks of fault detection and identification. Meanwhile, the ResNet with SENet and ResNet with DRSN only performed well in fault localization when the amount of data was large and did not maintain their advantages when the amount of data was small. However, the ResNet with CBAM achieved the highest classification accuracy for the three diagnostic tasks on Ds3, which had a small amount of data. Therefore, the adopted CBAM enhanced the 2D spatial feature extraction capability of the 2D encoder.

### 5.2. Multi-Dimensional Feature Extraction vs. Single-Dimension Feature Extraction

The advantages of the proposed multi-dimensional feature extraction method are discussed in this section. In this experiment, the multi-dimensional feature extraction method with the 2D and 1D encoders was trained. On three datasets, the average fault diagnosis accuracies of the 2D feature extraction consisting of Resnet and different attention modules, the 1D feature extraction based on Bi-LSTM, and the multi-dimensional extraction consisting of the 1D and 2D encoders were compared. The results are summarized in Table 4. The average classification accuracy for all three diagnostic tasks is improved by adding the Bi-LSTM to the ResNet and CBAM combination. 

In terms of the average accuracy across the three datasets, the multi-dimensional feature extraction method outperforms the combination of ResNet and CBAM, which is the best-performing single-dimensional feature extraction method, by 0.04% and 0.02% in fault detection and identification, with accuracies of 99.64% and 99.62%, respectively. In fault localization, although the combination of ResNet and SENet performs well among the single-dimensional methods, it still lags behind the multi-dimensional methods by 0.06%. Additionally, it can be noticed that Bi-LSTM does not perform well on the three diagnostic tasks compared to other single-dimensional methods. However, the highest diagnostic accuracy was achieved when using Bi-LSTM as the 1D encoder and the combination of ResNet and CBAM as the 2D encoder for multi-dimensional feature extraction.

### 5.3. Multi-Task Learning vs. Single-Task Learning

This section describes the verification of the effectiveness of the proposed multi-task learning structure. To demonstrate that the multi-task learning structure can enhance the classification accuracy of the proposed model on the three fault diagnosis tasks, a comparative experiment between multi-task and single-task learning was conducted. The average diagnostic accuracies of the proposed multi-task model and single-task learning models for fault detection, fault identification, and fault localization on Ds1, Ds2, and Ds3 are listed in Table 5. The single-task learning structure for each classification task in the experiments was identical to that of the corresponding branch in the multi-task learning structure.

It can be seen from Table 5 that multi-task learning enables the model to achieve outstanding classification accuracy in multiple diagnostic tasks simultaneously. Although multi-task learning does not perform as well as single-task learning in fault localization, it outperforms single-task learning in terms of fault detection and identification. This finding further demonstrates that multi-task learning can increase the accuracy in multiple classification tasks.

By comparing the experimental results, the specific parameters of the proposed MAM-Net model were obtained; they are listed in Table 6.

### 5.4. Model Validation

#### 5.4.1. Compared Methods

To further demonstrate the superiority of the proposed MAM-Net method, a comparative experiment was performed using the existing fault diagnosis methods. These existing methods include single-dimensional feature extraction, multi-dimensional feature extraction, single-task learning, and multi-task learning approaches. We adjusted certain parameters of these models to align their input–output structures with the experiments of this study and fine-tuned certain model parameters using insights gained from multiple experiments in order to enhance the comparability of each model. Considering that the amount of fault data is small in practice, Ds3 was used as the dataset to train the models in the experiments described in this section. The parameters or structures of each model were as follows.

(1) MLP

This model consisted of linear layers, and the ReLU activation function was added between layers to improve the generalization ability of this model [62]. The structure of the MLP was as follows: {Input (75 × 14), linear (75 × 14, 512), linear (512, 256), linear (256, Class)}.

(2) LeNet

This model consisted of a combined stack of convolution and max pooling layers. The structure of LeNet was as follows: {Input (1, 75, 14), convolution (1, 64, 3), max pool (2, 2), convolution (64, 128, 3), max pool (2, 2), linear (1024, 512), linear (512, Class)} [28].

(3) DenseNet

This model consisted of multiple dense blocks containing a CNN. The network could alleviate the gradient problem caused by the deepening of the network by considering the output of all of the previous layers as the input into a deeper layer. The parameters of DenseNet were as follows: {Input (1, 75, 14), init_channels = 64, growth rate = 2, blocks = [6, 12, 24, 16], dropout = (0.5), linear (4352, 1024), linear (1024, 512), dropout (0.5), linear (512, Class)}.

(4) RepVGG

RepVGG was based on the VGG network and introduced a ResNet-like branching structure that improved the speed and accuracy of the network [39]. The parameters of the model were as follows: {Input (1, 75, 14), blocks = [1, 2, 4, 14, 1], width multiplier = [1, 1, 1, 2.5], dropout = (0.5), linear (512 × 2.5, 256), dropout (0.5), linear (256, Class)}.

(5) CNN

CNN are commonly used as models in DL. In this study, the CNN structure consisted of a convolutional layer and maximum pooling layer, whose details were as follows: {Input (1, 75, 14), convolution (1, 64, 3, 1, 1), max pool (3, 2, 1), convolution (64, 128, 3, 1, 1), max pool (3, 2, 1), convolution (128, 256, 3, 1, 1), max pool (3, 2, 1), GAP (1, 1), linear (5120, 512), dropout (0.5), linear (512, Class)}.

(6) Inception

Inception is a network model that can capture more feature information by simultaneously using multiple convolutional kernels of different sizes for feature extraction from the data [22]. In this study, the parameters of Inception were as follows: {Input (1, 75, 14), inception type = V2, dropout = (0.5), linear (1024, 512), dropout (0.5), linear (512, Class)}.

(7) CNN-LSTM

This type of model consists of a parallel network structure of CNN and LSTM models, using CNN and LSTM to extract temporal dimensional features simultaneously [37]. In this study, the following structure was used: {the CNN had the same structure as above, LSTM (14, 64), concatenate (256, 128), dropout (0.5), LSTM (64, 128), linear (256 + 128, 128), dropout (0.5), linear (128, Class)}.

(8) MFSMTP

MFSMTP is a multi-task learning network that can extract features from data using three different sizes of convolutional kernels to capture more feature information [32]. The parameters of MFSMTP were as follows: {Input (1, 75, 14), convolution1 (5, 1, 2), convolution2 (3, 1, 1), convolution3 (1, 1, 0), linear (192, Class)}.

(9) MTL-CNN

MTL-CNN is a multi-task learning model that takes a traditional CNN as the main body of feature extraction. This approach enables the model to maintain both rapid diagnosis speed and high classification accuracy when addressing multiple diagnostic tasks [63]. The structure of MTL-CNN was as follows: {Convolution (1, 64, 3, 1, 1), convolution (1, 64, 3, 1, 1), convolution (1, 64, 3, 1, 1), max pool (3, 2, 1), convolution (1, 64, 3, 1, 1), convolution (1, 64, 3, 1, 1), max pool (3, 2, 1), linear (228, 128), linear (128, class)}.

The Precision, Recall, and *F*1 score were used to evaluate and compare the fault diagnosis performances of different models [64]. The formulae for these evaluation metrics are as follows:

(7) $$Precision=\frac{TP}{TP+FP},$$

(8) $$Recall=\frac{TP}{TP+FN},$$

(9) $$F1=\frac{2\times Precision\times Recall}{TP+FP},$$

where *TP* and *FP* refer to the numbers of samples correctly and incorrectly classified as positive, respectively, and *TN* and *FN* refer to the numbers of samples correctly and incorrectly classified as negative, respectively.

#### 5.4.2. Comparison of Fault Detection Performance

In this experiment, we compared the three metrics of the proposed model with other methods in terms of fault detection. Table 7 lists the fault diagnosis performance of the different models after 50 training epochs. The best values of each metric are highlighted in bold. The number of epochs was set at 50 based on the observation that the proposed model and other comparative models have achieved stability in terms of loss and classification accuracy.

The results show that the proposed model is superior to the other models in all fault detection metrics. MAM-Net outperforms MSFMTP, which is the best performer among the other models, in terms of Precision, Recall, and *F*1 by 0.71%, 1.76%, and 1.25%, respectively. It is worth noting that although MTL-CNN, MSFMTP, and MAM-Net are all multi-task learning methods, the proposed model outperforms MTL-CNN in all three metrics by a margin of 5.33%, 3.64%, and 4.55%, respectively. This is because MTL-CNN can only extract features from a single dimension at a fixed scale. Although MSFMTP can utilize multi-scale extraction to obtain more feature information, its accuracy is still lower than the proposed model MAM-Net due to the limitation of its extraction dimension.

**Figure 9:**
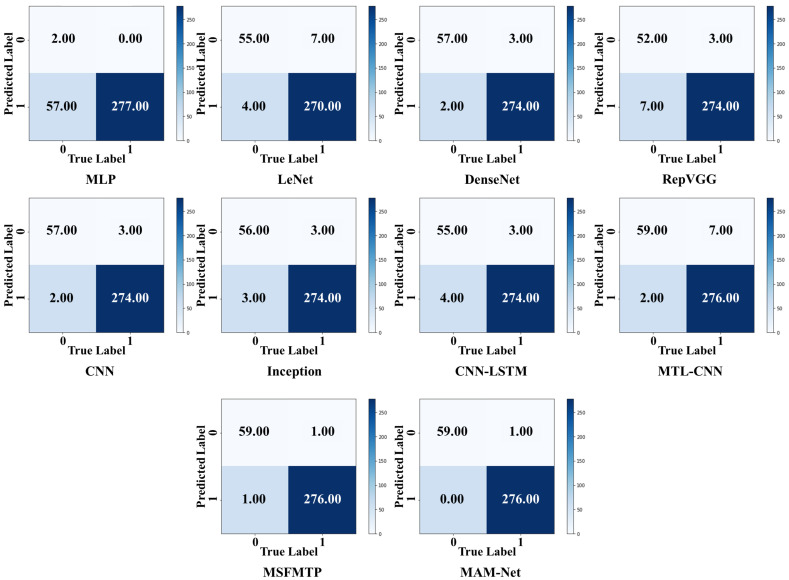
Confusion matrices of different fault detection methods.

To visually display the classification results, a confusion matrix was introduced as a visualization tool, as shown in Figure 9. Evidently, the proposed MAM-Net model can predict the fault states of the samples with minimal error. In contrast, the other models exhibit confusion between the fault and health states during fault detection, especially the MLP, which makes accurate fault state classification difficult.

#### 5.4.3. Comparison of Fault Identification Performance

As shown in Table 7, for fault type identification, MAM-Net has a higher classification accuracy than the other models. DenseNet performed well in fault type identification; however, it was still outperformed by MAM-Net in each metric. In terms of Precision, MAM-Net performed 0.94% better than DenseNet. In terms of Recall and F1, the proposed method exceeded DenseNet by 1.09% and 1.03%, with values of 99.80% and 99.81%, respectively. 

**Figure 10:**
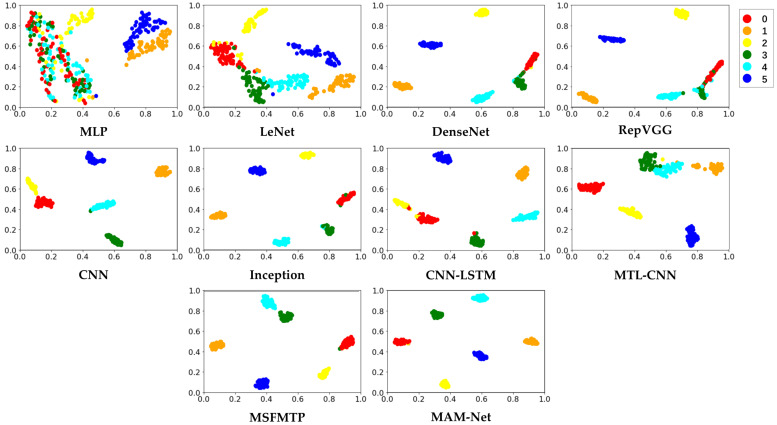
T-SNE visualization for different fault identification methods. 0 represents no fault, and 1, 2, 3, 4, and 5 represent break fault, bias fault, spike fault, noise fault, and gain fault, respectively.

For a clearer comparison of the classification results of the different methods, T-SNE was introduced to downscale the high-dimensional feature maps extracted by the model to a 2D space, as shown in Figure 10. Evidently, the proposed method can easily classify and aggregate samples from different fault types. By contrast, MLP and LeNet performed poorly in distinguishing the fault classes. In the other methods, although models such as DenseNet, RepVGG, CNN, and MTL-CNN can cluster some fault types, there is still no clear boundary between certain fault types. Models such as Inception, CNN-LSTM, and MSFMTP are able to make a relatively clear distinction between each fault type by capturing multiple features simultaneously, but there are still some samples that are confused.

#### 5.4.4. Comparison of Fault Localization Performance

**Figure 11:**
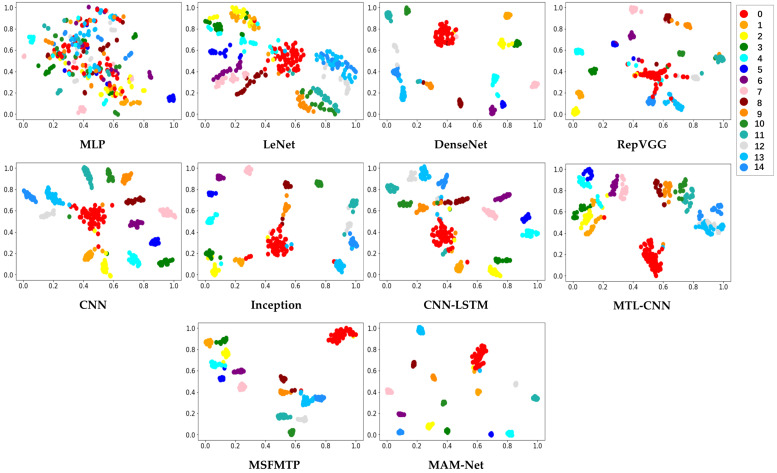
T-SNE visualization results obtained using different methods for fault localization, where 0 represents no sensor with a fault, and 1–14 represent the 14 sensors with faults.

Fault location information also plays an important role in fault diagnosis. The data in Table 7 and T-SNE visualization results in Figure 11 emphasize the strength of the proposed framework in fault localization. As shown in Table 7, CNN, LeNet, MSFMTP, and the proposed MAM-Net all perform well in fault localization. The highest classification accuracy is still achieved by MAM-Net. Figure 11 shows that the intra-class shrinkage of MLP, CNN, LeNet, and MTL-CNN is worse than the other methods. This finding indicates that several models with better classification results may exhibit decreases in accuracy as data complexity increases. However, the MAM-Net method proposed in this study can increase the inter-class distance well to distinguish each location and also shorten the intra-class distance effectively to aggregate samples with the same fault location.

## 6. Discussion

### 6.1. Diagnostic Performance of Different Methods on Different Amounts of Data

Fault data are typically difficult to collect when a fault diagnosis model is applied in practice, such that the number of samples within the training dataset is small. Therefore, the classification performances of each method on Ds3, which contained a small amount of data, were compared in the model validation experiments described in Section 5.4. The classification accuracies of deep learning models usually improve as the amount of data in the training set increases [65]. Therefore, the advantages of the proposed MAM-Net model for the three diagnostic tasks when the amount of data increases were verified as described in this section. The settings of the experiments in this section are the same as those described in Section 5.4.1 for the experiments conducted on Ds3. The performances of each model on Ds1 and Ds2, two datasets containing more data than Ds3, are presented in Table 8.

As shown in Table 8, the best performance of the proposed model is reflected in fault detection, identification, and localization, with accuracies of 98.39%, 99.46%, and 99.75%, respectively, when trained on Ds2 with a moderate amount of data. For Ds1, which has a large amount of data, the classification accuracy performances of the other models are close to that of MAM-Net but still lower in terms of overall performance. For example, MAM-Net has the highest accuracy of 99.99% for fault localization, and RepVGG achieves the same accuracy as the proposed model (99.92%) for fault identification. Although the proposed method lags behind Inception in fault detection, it still outperforms Inception in the other classification tasks. Therefore, the solution proposed in this study can achieve better performance with different amounts of data.

### 6.2. Diagnostic Performance of MAM-Net on Imbalanced Dataset

To achieve accurate diagnostic predictions, balanced data are required to train deep neural networks. However, the collection of faulty samples in some cases is not easy. Therefore, the problem of imbalanced training data samples exists. Imbalanced data samples make training a model to achieve accurate sensor fault diagnosis relatively difficult. This section examines the fault diagnosis effectiveness of the proposed MAM-Net method for imbalanced samples. The dataset used here was the original dataset without balancing the number of samples at each gas concentration; that is, the number of fault samples was not the same for different gas concentrations. Table 9 lists the fault diagnosis accuracies of the models for the unbalanced dataset. From the table, it can be seen that the average accuracies of the proposed model for fault detection and localization are 99.74% and 99.90%, respectively. Compared to the highest accuracies obtained among the other methods, MAM-Net achieves accuracy improvements of 0.53% and 0.11% for fault detection and localization, respectively. For fault identification, the classification accuracy of the proposed model is 99.78%, which is only 0.06% below the highest accuracy. Therefore, MAM-Net still outperforms the other methods in terms of overall performance on the imbalanced dataset.

### 6.3. Generalization Performance of MAM-Net

In the practical application of fault diagnosis models, in addition to the importance of fault diagnostic accuracy, the generalizability of the model (i.e., consistently excellent performance across different data sources) should also be considered. Therefore, to test the generalization of the proposed model on different datasets, a dataset of gas mixtures collected by Fonollosa [66] was used for the experiments described in this section. The dataset was obtained from 16 MOX gas sensors exposed to the two gas mixtures for 12 h of continuous measurements. The two gas mixtures were ethylene and methane in air and ethylene and carbon monoxide in air.

The experiments discussed in this section were started by using the same method to preprocess the data as described above, and the sample size obtained was 75 × 16 (where 75 denotes the length of the sampling period, and 16 denotes the number of sensors). Subsequently, fault injection was applied to generate the fault dataset and divide the obtained fault dataset into training, validation, and testing datasets in the same ratio. Finally, the generalization of the proposed MAM-Net method was verified by training each model on these datasets and comparing their accuracies on the three classification tasks. Table 10 shows the classification accuracies of each model in the three fault diagnosis tasks.

In Table 10, similar to the situation for the previous dataset, the proposed method exhibits the best overall performance in the three classification tasks. The accuracy of MAM-Net in fault identification reaches 98.70%, exceeding that of the CNN, which was the best among the other methods, by 1.08%. It also surpassed the CNN in fault localization performance by 0.12%. In fault detection, although not the best, the proposed approach achieves an accuracy only 0.09% away from the highest accuracy. Therefore, the proposed MAM–Net model provides good generalization and enables the application of gas sensor data in different situations for fault diagnosis. 

### 6.4. Real-Time Analysis of the Proposed Methods

In practical implementations of fault diagnosis systems, beyond the essential criteria of accuracy, it is critical to take into account the model’s diagnostic time for gas-based real-time fault detection. The prediction time of the model must be sufficiently short to meet the requirements of real-time diagnosis. We recorded the time (in seconds) taken by each method to perform prediction on a batch of test dataset to reflect the computational burden, as the computation time for a single sample is exceptionally short. The prediction model used was obtained through 50 epochs of training on Ds3. The recorded times are presented individually and summarized in Table 11.

In the experiment, the carbon monoxide gas sensor dataset had a heating period of 25 s and a sampling rate of 0.333 s. The data cycle for fault diagnosis was based on the sensor heating cycle. For example, to accurately distinguish between different fault types within a sample period, such as distinguishing spike faults from noise faults, a heating cycle is necessary to obtain the characteristics of this fault. We record the time (in seconds) taken by each method to perform prediction on a batch of test data to reflect the computational burden, as the computation time for a single sample is exceptionally short. From Table 11, it can be observed that its diagnostic time (0.462554 s for a batch, i.e., 0.01445 s for one sample) is far less than the duration of a single sample cycle and a sampling rate of 0.333 s. Therefore, the proposed MAM-Net method can meet the real-time requirements of practical applications while achieving high diagnostic accuracy.

## 7. Conclusions

In this study, we developed a novel gas sensor array fault diagnosis method called MAM-Net based on multi-dimensional feature fusion, an attention mechanism, and multi-task learning. The multi-dimensional feature fusion module can obtain sufficient and effective diagnostic feature information from sensor array data by integrating fault feature representations from different dimensions. With this module, the diagnostic performance of the model can be improved by providing better feature capture capabilities. The multi-task learning module developed in this study can simultaneously perform classification tasks for fault detection, identification, and localization. The module can integrate and supplement more diagnostic information through parameter sharing between multiple tasks to improve the fault diagnostic accuracy of the model further. The experimental results show that the MAM-Net method is significantly better than other methods in terms of classification metrics and result visualization for fault diagnosis with different amounts of data, different experimental settings, and balanced and unbalanced datasets. 

In future work, considering the requirement of real-time fault diagnosis for various gas-based applications, we will focus on lightening the structure of the proposed model to shorten the diagnosis time and minimize the computational burden. Meanwhile, we will conduct further research on data reconstruction strategies for faulty sensors based on GAN methods.

## Figures and Tables

**Table 1:** Settings for each type of fault.

Fault Type	K Value	$B\left(t\right)$ Value
Healthy signal	1	0
Broken circuit fault	0	0
Bias fault	0	(0.95–1.05)$\;\times \;x\left(a\right)$, a: time of failure
Spike fault	1	40–45 (random variations in the cycle)
Noise fault	0.6–1.4, varies with time	0
Gain fault	1.5–2.0, varies with time	0

**Table 2:** Number of various types of faults and health signals in fault datasets. The “8” in the table represents the eight gas concentration cases.

Fault Type	Label	Ds1	Ds2	Ds3
Healthy signal	0	2128 (266 × 8)	1120 (140 × 8)	560 (70 × 8)
Broken circuit signal	1	2128	1120	560
Bias fault	2	2128	1120	560
Spike fault	3	2128	1120	560
Noise fault	4	2128	1120	560
Gain fault	5	2128	1120	560

**Table 3:** Acc of ResNet with different Dep and Dim.

	Model	Acc (%) (Fault Detection)			Acc (%) (Fault Identification)			Acc (%) (Fault Localization)		
		Ds1	Ds2	Ds3	Ds1	Ds2	Ds3	Ds1	Ds2	Ds3
Dep	ResNet18	99.73	97.87	**98.93**	99.88	99.16	99.04	99.97	99.76	99.28
	ResNet34	**100.0**	98.39	98.52	99.92	**99.46**	99.30	**99.98**	**99.78**	**99.56**
	ResNet50	100.0	**98.49**	98.38	**99.98**	99.28	**99.41**	99.98	99.77	99.24
Dim	1D-ResNet 34	99.86	**98.82**	94.03	99.77	99.40	97.10	99.79	99.40	95.25
	2D-ResNet 34	**100.0**	98.39	**98.52**	**99.92**	**99.46**	**99.30**	**99.98**	**99.78**	**99.56**

The best values for each experiment are in bold.

**Table 4:** Diagnostic accuracies of different attentional modules under single-dimensional feature extraction methods and the diagnostic accuracy of the multi-dimensional feature extraction method.

Method	Model	Acc (%) (Fault Detection)			Acc (%) (Fault Identification)			Acc (%) (Fault Localization)		
		Ds1	Ds2	Ds3	Ds1	Ds2	Ds3	Ds1	Ds2	Ds3
Single-dimensional feature extraction	ResNet + SENet	100.0	99.20	99.08	99.86	99.39	98.71	**99.97**	99.49	99.51
	ResNet + DRSN	99.98	98.78	98.67	99.77	99.30	98.50	99.80	**99.58**	99.36
	ResNet + CBAM	**100.0**	**99.66**	**99.14**	**99.90**	**99.41**	**99.50**	99.86	99.30	**99.56**
	Bi-LSTM	97.75	78.21	78.68	95.65	83.14	79.65	96.69	82.99	75.99
Multi-dimensional feature extraction	ResNet(CBAM) + Bi-LSTM	99.97	**99.69**	**99.25**	**99.92**	99.40	**99.54**	99.96	99.38	**99.82**

The best values for each experiment are in bold.

**Table 5:** Diagnosis accuracies of single- and multi-task learning.

Method	Acc (%) (Fault Detection)			Acc (%) (Fault Identification)			Acc (%) (Fault Localization)		
	Ds1	Ds2	Ds3	Ds1	Ds2	Ds3	Ds1	Ds2	Ds3
Single fault detection	99.54	97.37	97.37	-	-	-	-	-	-
Single fault diagnosis	-	-	-	99.85	99.26	99.26	-	-	-
Single fault localization	-	-	-	-	-	-	**99.98**	**99.77**	99.77
Multi-task learning	**99.97**	**99.69**	**99.25**	**99.92**	**99.40**	**99.54**	99.96	99.38	**99.82**

The best values for each experiment are in bold.

**Table 6:** Details of the proposed model.

Module	Layer	Specification	Output Size
--	Inputs	--	1×75×14
2D encoder ResNet34 (CBAM)	Conv1	3 × 3, 64, s =1, p = 1	64×75×14
	Max pool	3 × 3, s =2, p = 1	64×38×7
	Conv2_x	$\left[\begin{array}{cc} 3\times 3, & 64\\ 3\times 3, & 64 \end{array}\right]\times 3$	64×38×7
	Conv3_x	$\left[\begin{array}{cc} 3\times 3, & 128\\ 3\times 3, & 128 \end{array}\right]\times 4$	128×19×4
	Conv4_x	$\left[\begin{array}{cc} 3\times 3, & 256\\ 3\times 3, & 256 \end{array}\right]\times 6$	256×10×2
	Conv5_x	$\left[\begin{array}{cc} 3\times 3, & 512\\ 3\times 3, & 512 \end{array}\right]\times 3$	512×5×1
	Gap	Output size = (1,1)	512×1×1
1D encoder	Bi-Lstm	Hidden size = 256	512×1
Multi-task learning	Linear	(1024, 512)	512×1
	Linear	(512, Class)	Class×1

**Table 7:** Diagnostic performance of each model.

Methods	Fault Detection (%)			Fault Identification (%)			Fault Localization (%)		
	Precision	Recall	*F*1	Precision	Recall	*F*1	Precision	Recall	*F*1
MLP	77.51	53.88	53.00	68.44	66.18	66.37	89.70	61.77	70.27
LeNet	95.36	96.56	95.95	95.39	94.70	94.90	97.31	95.62	96.38
DenseNet	95.89	95.89	95.90	98.89	98.71	98.78	96.14	94.94	95.47
RepVGG	94.61	96.37	95.50	94.96	94.79	94.72	91.76	91.34	91.44
CNN	97.31	96.92	97.09	98.72	98.64	98.66	99.31	99.00	99.14
Inception	95.27	93.68	94.44	96.68	96.53	96.52	92.09	92.80	92.20
CNN-LSTM	96.31	96.06	96.18	98.34	98.17	98.25	95.26	94.05	94.53
MTL-CNN	94.08	96.23	95.10	92.04	92.05	92.00	82.69	81.17	81.49
MSFMTP	98.70	98.11	98.40	97.23	98.20	97.16	97.68	97.31	97.41
Proposed model (MAM-Net)	**99.41** **(↑0.71)**	**99.87** **(↑1.76)**	**99.65** **(↑1.25)**	**99.83** **(↑0.94)**	**99.80** **(↑1.09)**	**99.81** **(↑1.03)**	**99.86** **(↑0.55)**	**99.69** **(↑0.69)**	**99.78** **(↑0.64)**

The best values of each metric are in bold, ↑ represents the improvement achieved by the proposed model compared to the other models with the highest accuracy.

**Table 8:** Diagnostic accuracies of each model on different amounts of data.

Method	Acc (%) (Fault Detection)		Acc (%) (Fault Identification)		Acc (%) (Fault Localization)	
	Ds1	Ds2	Ds1	Ds2	Ds1	Ds2
MLP	80.52	74.01	73.56	69.91	86.21	85.43
LeNet	98.60	93.83	99.70	97.90	99.71	97.88
DenseNet	99.51	94.96	99.77	98.21	99.94	98.85
RepVGG	99.77	96.02	99.92	98.24	99.98	97.83
CNN	99.58	97.17	99.76	99.26	99.86	99.48
Inception	**99.95**	97.38	99.49	98.27	99.49	96.72
CNN-LSTM	99.17	97.24	99.60	99.45	99.67	98.53
MTL-CNN	99.54	95.00	98.79	95.30	99.93	92.78
MSFMTP	99.73	97.25	99.92	98.56	99.92	99.12
Proposed model (MAM-Net)	99.77 (–)	**98.39 (↑1.01)**	**99.92 (–)**	**99.46 (↑0.01)**	**99.99 (↑0.01)**	**99.75 (↑0.27)**

The best values for each experiment are in bold, ↑ represents the improvement achieved by the proposed model compared to the other models with the highest accuracy; – represents no improvement.

**Table 9:** Diagnostic accuracies of each model on the imbalanced dataset.

Method	Acc (%) (Fault Detection)	Acc (%) (Fault Identification)	Acc (%) (Fault Localization)
MLP	78.43	74.78	83.62
LeNet	88.12	99.41	99.77
DenseNet	99.21	99.44	99.51
RepVGG	99.09	99.62	99.77
CNN	99.21	**99.84**	99.79
Inception	99.07	99.65	99.43
CNN-LSTM	98.78	94.05	94.53
MTL-CNN	96.41	92.25	95.99
MSFMTP	97.35	93.02	98.12
Proposed model (MAM-Net)	**99.74 (↑0.53)**	99.78 (–)	**99.90 (↑0.11)**

The best values for each experiment are in bold, ↑ represents the improvement achieved by the proposed model compared to the other models with the highest accuracy, – represents no improvement.

**Table 10:** Diagnostic accuracies of each method on the dataset from [66].

Method	Acc (%) (Fault Detection)	Acc (%) (Fault Identification)	Acc (%) (Fault Localization)
MLP	78.10	58.80	75.78
LeNet	86.08	86.23	96.55
DenseNet	96.70	96.79	98.28
RepVGG	94.31	97.16	98.80
CNN	98.85	97.62	99.38
Inception	98.21	96.97	98.74
CNN-LSTM	**99.09**	97.54	98.86
MTL-CNN	98.57	92.38	97.48
MSFMTP	98.24	92.87	98.04
Proposed model (MAM-Net)	99.00 (–)	**98.70 (** **↑** **1.08)**	**99.50 (** **↑** **0.12)**

The best values for each experiment are in bold, ↑ represents the improvement achieved by the proposed model compared to the other models with the highest accuracy, – represents no improvement.

**Table 11:** Diagnostic time for a batch of samples on different diagnostic tasks.

Method	Diagnostic Time (s) (Fault Detection)	Diagnostic Time (s) (Fault Identification)	Diagnostic Time (s) (Fault Localization)
MLP	0.000908	0.000908	0.001090
LeNet	0.021080	0.024532	0.021260
DenseNet	0.108900	0.101667	0.116933
RepVGG	0.093128	0.078500	0.095940
CNN	0.566950	0.051789	0.069959
Inception	0.072141	0.067871	0.065235
CNN-LSTM	0.127745	0.131743	0.133921
MSFMTP	0.079318 (Simultaneous for three tasks)		
MTL-CNN	0.134287 (Simultaneous for three tasks)		
Proposed model (MAM-Net)	0.462554 (Simultaneous for three tasks)		

## Data Availability

Not applicable.
